# Baseline Cardiovascular Risk Factor Control in Patients With Type 2 Diabetes and Coronary Disease Versus Stroke: Secondary Analysis of Cardiovascular Outcome Trials

**DOI:** 10.1161/STROKEAHA.122.042053

**Published:** 2023-07-14

**Authors:** Priyadarshini Balasubramanian, Walter N. Kernan, Kevin N. Sheth, Anne Pernille Ofstad, Julio Rosenstock, Christoph Wanner, Bernard Zinman, Michaela Mattheus, Nikolaus Marx, Silvio E. Inzucchi

**Affiliations:** Section of Endocrinology, Department of Medicine (P.B.), Yale School of Medicine, New Haven, CT.; Section of General Internal Medicine, Department of Medicine (W.N.K), Yale School of Medicine, New Haven, CT.; Department of Neurology (K.N.S), Yale School of Medicine, New Haven, CT.; Section of Endocrinology, Department of Medicine (S.E.I), Yale School of Medicine, New Haven, CT.; Boehringer Ingelheim Norway KS, Asker (A.P.O.).; Oslo Diabetes Research Center, Norway (A.P.O.).; Velocity Clinical Research at Medical City, Dallas, TX (J.R.).; Würzburg University Clinic, Germany (C.W.).; Lunenfeld-Tanenbaum Research Institute, Mount Sinai Hospital, University of Toronto, ON, Canada (B.Z.).; Boehringer Ingelheim Pharma GmbH & Co KG, Germany (M.M.).; Department of Internal Medicine, University Hospital; RWTH Aachen University, Germany (N.M.).

**Keywords:** anticoagulant, cardiovascular disease, hypertension, odds ratio, risk factors

## Abstract

Patients with type 2 diabetes (T2D) and cardiovascular disease are at increased risk for recurrent ischemic events. Cardiovascular risk factor control is vital for secondary prevention, but how this compares among individuals with different T2D macrovascular complications is unknown. We aimed to determine if there might be differences in risk factor control in patients with T2D with previous stroke versus coronary artery disease (CAD).

**METHODS:**

Cross-sectional analyses were performed on 12 856 patients with T2D with prior history of stroke with or without CAD from 3 diabetes cardiovascular outcome trials: CARMELINA (The Cardiovascular and Renal Microvascular Outcome Study With Linagliptin), EMPA-REG OUTCOME (Empagliflozin Cardiovascular Outcome Event Trial in Type 2 Diabetes Mellitus Patients), and CAROLINA (The Cardiovascular Outcome Study of Linagliptin vs Glimepiride in Type 2 Diabetes). Risk factors at baseline assessed included dyslipidemia, hypertension, smoking, and current antiplatelet/anticoagulant therapy. Control, respectively, was defined as LDL (low-density lipoprotein)-C <100 mg/dL or statin use, systolic blood pressure <140 and diastolic blood pressure <90 mm Hg, not currently smoking, and use of an antiplatelet/anticoagulant medication. The odds ratio of 3 to 4 (or good) versus 0 to 2 (or suboptimal) risk factors controlled was analyzed by logistic regression models.

**RESULTS:**

The odds for good versus suboptimal risk factor control in patients with CAD alone was higher than in those with stroke alone across all 3 trials odds ratios (95% CI): CARMELINA, 2.05 (1.67–2.51), EMPA-REG OUTCOME, 2.50 (2.10–2.99), and CAROLINA, 1.63 (1.21–2.20). The respective odds ratios were lower (and rendered nonsignificant in CAROLINA) when cardiovascular risk factor control in patients with both CAD and stroke were compared with those with stroke alone: CARMELINA, 1.45 (1.13–1.87); EMPA-REG OUTCOME, 1.62 (1.25–2.08); and CAROLINA, 1.16 (0.74–1.83).

**CONCLUSIONS:**

In contemporary populations of patients with T2D, there was significant discordance in control of cardiovascular risk factors between patients with stroke versus CAD, with the former having less optimal control. The intermediate results in patients with both CAD and stroke suggest that these differences could be related at least in part to clinician factors.

**REGISTRATION:**

URL: https://www.clinicaltrials.gov; Unique identifiers: NCT01243424, NCT01131676, NCT01897532.

Ischemic stroke is a leading cause of morbidity and mortality world wide and contributes significantly to health care expenditures. Type 2 diabetes (T2D) is common among patients with stroke and is a major risk factor for recurrent stroke events. During the last few decades, research has shown that the recurrence risk may be reduced by careful attention to specific interventions including lipid lowering, management of hypertension, smoking cessation, and antiplatelet therapy. Despite these advances the risk of recurrent stroke remains as high as 15% to 30% in the first 5 years after an initial event.^1^ About 1 in 2 survivors remain with some form of disability and about 1 in 7 require institutionalized care.^2^ Preventing recurrent stroke as well as other atherosclerotic cardiovascular disease events is therefore a major goal of care for patients with cerebrovascular disease. There are few data, however, on the relative control of these risk factors during the outpatient care of patients after stroke versus myocardial infarction (MI) or, more broadly, coronary artery disease (CAD).

Over the past decade, large cardiovascular outcome trials in patients with T2D have demonstrated cardiovascular safety and efficacy of newer glucose-lowering medications. The regulatory mandate to assess the cardiovascular safety of new glucose-lowering medications stemmed from concerns related to older drugs used for T2D that may have actually increased cardiovascular risk.^3^ In the outcome trials conducted with the newer T2D medications, extensive information was obtained at baseline regarding prior cardiovascular diseases as well as control of atherosclerotic cardiovascular disease risk factors. This provided an opportunity to compare the degree of baseline cardiovascular risk factor control among patients with T2D with prior stroke and CAD. We specifically analyzed the results from 3, large cardiovascular outcome trials in patients with T2D, seeking to determine if there might be significant differences in the extent to control cardiovascular risk factors in patients with T2D with previous stroke versus CAD.

## METHODS

The data, analytic methods, and study materials will be made available to other researchers for purposes of reproducing the results or replicating the procedure. To ensure independent interpretation of clinical study results and enable authors to fulfil their role and obligations under the ICMJE criteria, Boehringer Ingelheim grants all external authors access to relevant clinical study data. In adherence with the Boehringer Ingelheim Policy on Transparency and Publication of Clinical Study Data, scientific and medical researchers can request access to clinical study data after publication of the primary article and secondary analyses in peer-reviewed journals and regulatory and reimbursement activities are completed, normally within 1 year after the marketing application has been granted by major Regulatory Authorities. Researchers should use the https://vivli.org/ link to request access to study data and visit https://www.mystudywindow.com/msw/datasharing for further information.

The article follows the STROBE (Strengthening the Reporting of Observational Studies in Epidemiology) reporting guidelines (Supplemental Material). Written informed consent was obtained from all participants in the original trials, per Good Clinical Practice guidelines. Given the nature of this study, no additional consent was warranted.

### Study Design

We performed a series of cross-sectional analyses of patient level data from 3 recent cardiovascular outcome trials in T2D: EMPA-REG OUTCOME (Empagliflozin Cardiovascular Outcome Event Trial in Type 2 Diabetes Mellitus Patients), CAROLINA (The Cardiovascular Outcome Study of Linagliptin vs Glimepiride in Type 2 Diabetes), and CARMELINA (The Cardiovascular and Renal Microvascular Outcome Study With Linagliptin), where we had complete access to participant-level baseline data. EMPA-REG OUTCOME was a multicenter randomized placebo-controlled trial that evaluated the efficacy and safety of the SGLT2 (sodium-glucose cotransporter-2) inhibitor empagliflozin in addition to standard of care in 7020 patients with T2D and established cardiovascular disease.^4^ CAROLINA was a multicenter, active-controlled, randomized clinical trial that studied cardiovascular outcomes in 6033 patients with T2D and high cardiovascular risk randomized 1:1 to linagliptin, a DPP-4 (dipeptidyl peptidase 4) inhibitor, or the sulfonylurea glimepiride when added to usual therapy.^5^ High cardiovascular risk was defined as documented atherosclerotic cardiovascular disease, multiple cardiovascular risk factors (at least 2 of the following: T2D duration >10 years; systolic blood pressure >140 mm Hg or receiving at least 1 blood pressure–lowering treatment; current smoker; low-density lipoprotein cholesterol ≥135 mg/dL [3.5 mmol/L] or receiving lipid-lowering treatment), aged at least 70 years, or evidence of microvascular complications (impaired kidney function [eGFR of 30–59 mL/min per 1.73 m^2^ or urine albumin/creatinine ratio ≥30 mg/g]) or proliferative retinopathy.^5^ CARMELINA was a third multicenter randomized placebo-controlled trial enriched with patients with evidence of chronic kidney disease that evaluated the effect of linagliptin versus placebo, when added to usual care, on cardiovascular and prespecified kidney outcomes in 6979 patients with T2D and high cardiovascular and renal risk. High cardiovascular risk and renal risk in CARMELINA was defined as history of macrovascular disease with urine albumin/creatinine ratio ≥30 mg/g; or reduced eGFR with or without urine albumin/creatinine ratio >200 mg/g.^6^

The major design features of the 3 trials have been shown in Table 1.

**Table 1.**
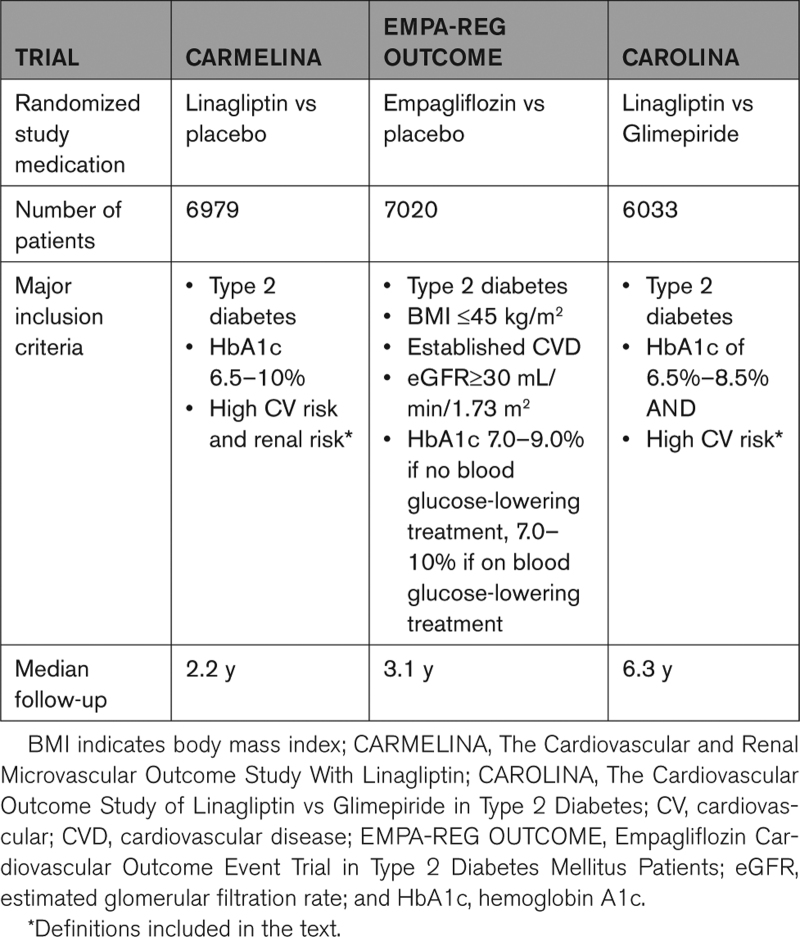
Major Design Characteristics of the 3 Trials

For the current exploratory analyses, we included participants from these 3 trials who had a history of stroke with or without CAD and available data on baseline cardiovascular risk factor control.

### Definition of Cardiovascular Disease Groups

Patients were defined as having a history of stroke or CAD based on investigator reports. CAD was defined as history of MI or coronary artery bypass graft or otherwise documented CAD. We defined 3 mutually exclusive groups for subsequent comparison: one that had a history of stroke only (ie, without CAD), 1 with a history of CAD only (ie, without stroke), and 1 that had a history of both stroke and CAD.

### Definition of Cardiovascular Risk Factor Control

Data on risk factors were captured at baseline. Risk factors assessed were dyslipidemia, hypertension, smoking, and use of antiplatelet and anticoagulant drugs. Control of a risk factor was defined as baseline (1) LDL (low-density lipoprotein) cholesterol <100 mg/dL or statin use, (2) systolic blood pressure <140 mm Hg and diastolic blood pressure <90 mm Hg, (3) not smoking, and (4) use of antiplatelet or anticoagulant drugs, respectively. In a sensitivity analysis, we additionally used LDL <70 mg/dL or statin use to define control of the lipid risk factor.

For all 3 studies, the information on characteristics to define the cardiovascular disease groups and cardiovascular risk factor control were mandatory to be collected as per each clinical trial protocol. Across all 3 studies, there was 1 patient in EMPA-REG OUTCOME with missing information on stroke, while all patients provided data to assess CAD. Among patients with CAD and stroke, 99.8% provided data for the assessment of cardiovascular risk factor control in EMPA-REG OUTCOME, 97.7% in CAROLINA, and 99.0% in CARMELINA.

### Statistical Analyses

Analyses were performed in each trial separately, based on patients randomized and treated with available data for cardiovascular disease groups and cardiovascular risk factor control and on pooled treatment arms within each trial. Baseline characteristics for cardiovascular disease groups were reported as means (continuous variables) or numbers and proportions (categorical variables) based on patients with data available for assessment of cardiovascular risk factor control.

The odds ratio (OD) of having all or most (3 to 4, and therefore good) versus less (0 to 2, and therefore suboptimal) cardiovascular risk factors controlled was analyzed by a logistic regression model that included a term for the cardiovascular disease group. We performed 2 comparisons: (1) patients with CAD alone versus stroke alone and (2) patients with both CAD and stroke versus patients with stroke alone. These analyses were repeated in subgroups by age, sex, and geographic region in separate logistic regression models including terms for subgroup and the interaction term of subgroup with cardiovascular disease group. In sensitivity analyses, we also assessed the control of 4 versus less (0 to 3) and 2 to 4 versus less (0 to 1) cardiovascular risk factors.

In further sensitivity analyses we performed adjustment for age, sex and region. All analyses were exploratory on a nominal 2-sided α=0.05 without adjustment for multiplicity. All statistical analyses were performed using SAS software, version 9·4 (Cary, NC.)

## RESULTS

The demographics and selected baseline characteristics by cardiovascular disease category for each of the 3 trials are shown in Table 2.

**Table 2.**
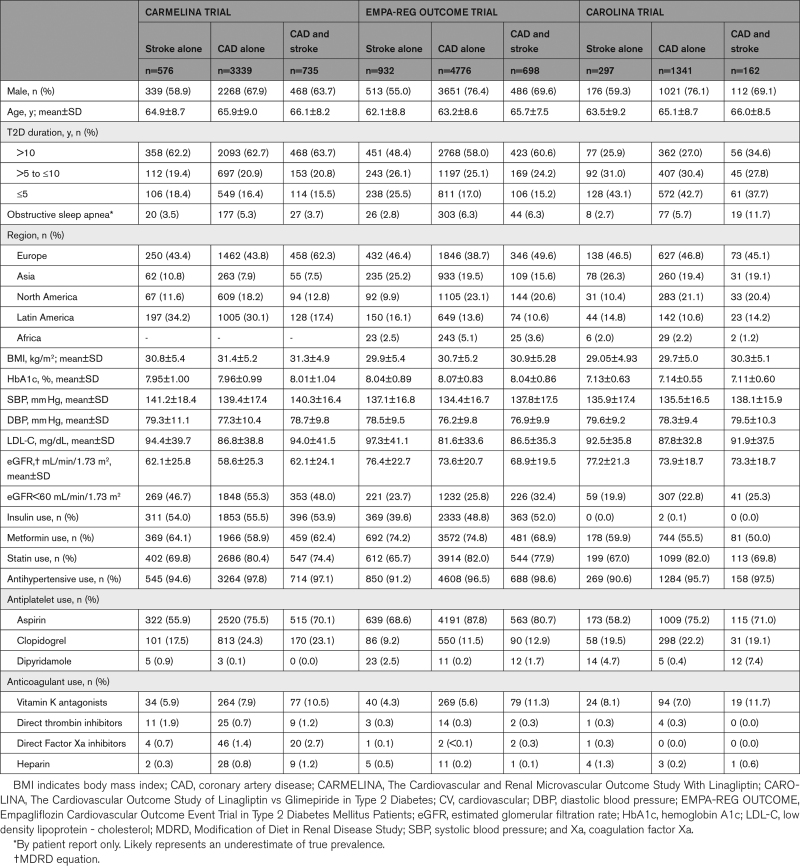
Baseline Characteristics by CV Disease Categories in the 3 Trials

**Figure 1.**
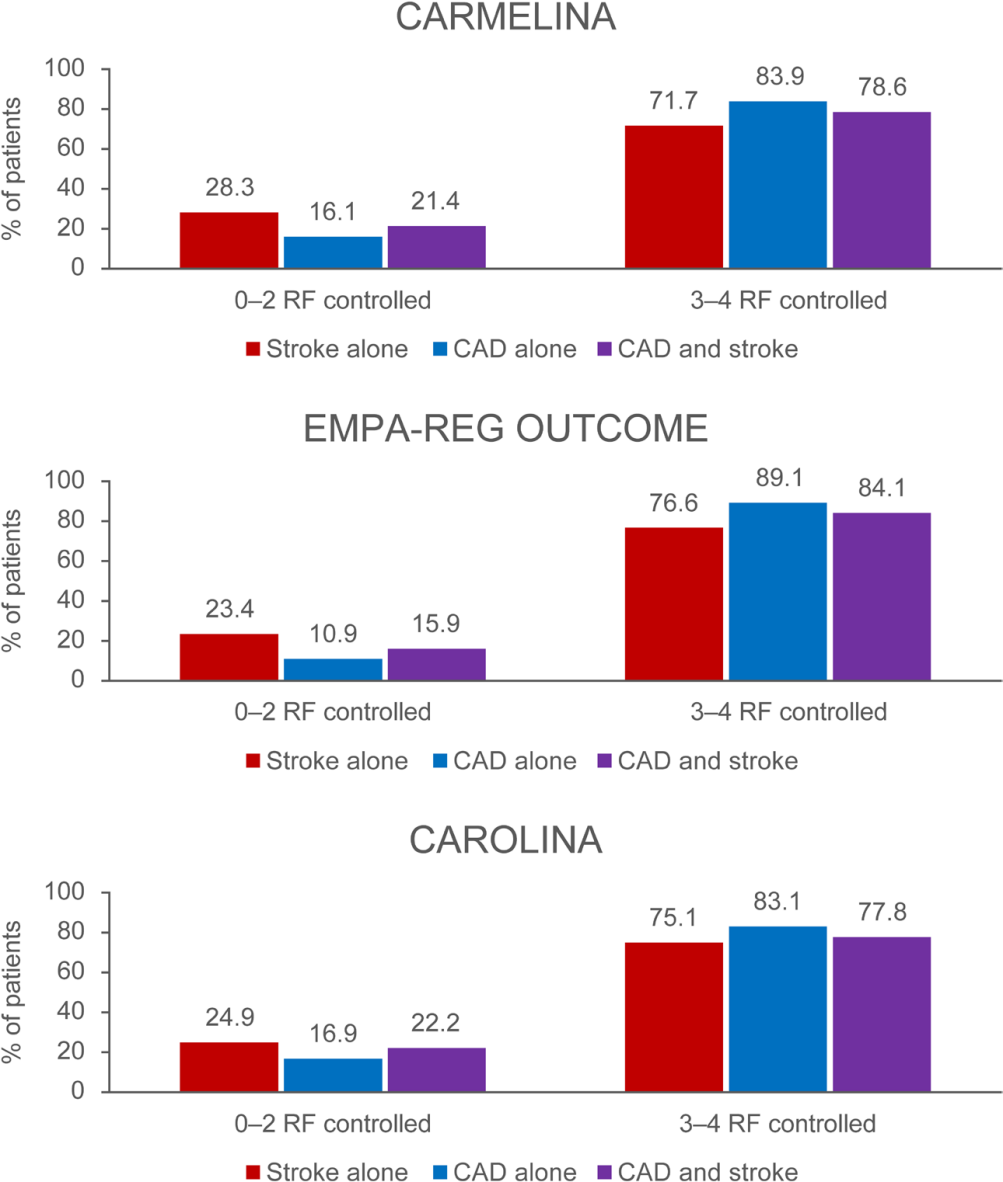
**The percentage of patients with good (3–4 risk factors) vs suboptimal (0–2 risk factors) controlled by cardiovascular disease subgroups at baseline in the 3 trials.** CAD indicates coronary artery disease; CARMELINA, The Cardiovascular and Renal Microvascular Outcome Study With Linagliptin; CAROLINA, The Cardiovascular Outcome Study of Linagliptin vs Glimepiride in Type 2 Diabetes; and EMPA-REG OUTCOME, Empagliflozin Cardiovascular Outcome Event Trial in Type 2 Diabetes Mellitus Patients.

Overall, 80.7% participants in the CARMELINA trial, 86.6% participants in EMPA-REG OUTCOME and 79.4% participants in the CAROLINA trial had 3 or 4 cardiovascular risk factors controlled at baseline. Among participants with CAD alone, 83.9% in the CARMELINA trial, 89.1% in the EMPA-REG OUTCOME trial and 83.1% in the CAROLINA trial had 3 or 4 cardiovascular risk factors controlled and the corresponding rates among participants with stroke alone were consistently lower at 71.7%, 76.6%, and 75.1%, respectively. When participants with both CAD and stroke were analyzed, the results appeared to be intermediate, at 78.6%, 84.1%, and 77.8% across the 3 trials, respectively. About 16.1% participants in CARMELINA trial, 10.9% participants in EMPA-REG OUTCOME, and 16.9% participants in CAROLINA trial had 0 to 2 risk factors controlled in the CAD alone group and the corresponding rates among participants with stroke alone were 28.3%, 23.4%, and 24.9%, respectively. Among patients with both CAD and stroke, 21.4%, 15.9%, and 22.2% had 0 to 2 risk factors controlled. The percentage of patients with good versus suboptimal cardiovascular risk factor control by cardiovascular disease groups is shown in Figure 1. Cardiovascular risk factor control by cardiovascular groups is shown in Table 2.

**Figure 2.**
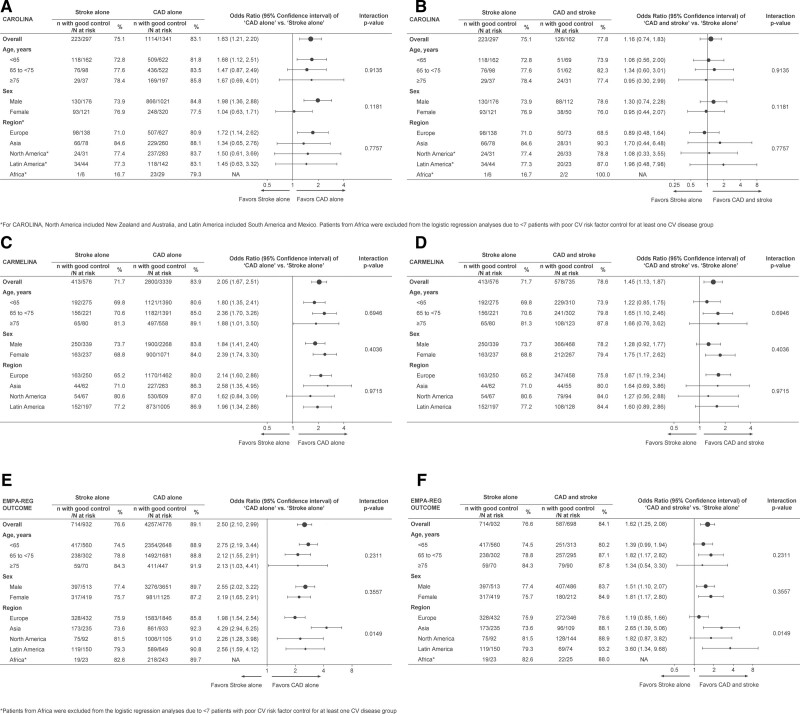
**Forest plot showing cardiovascular (CV) risk factor control by CV disease groups in the 3 trials.** CAD indicates coronary artery disease; CARMELINA, The Cardiovascular and Renal Microvascular Outcome Study With Linagliptin; CAROLINA, The Cardiovascular Outcome Study of Linagliptin vs Glimepiride in Type 2 Diabetes; and EMPA-REG OUTCOME, Empagliflozin Cardiovascular Outcome Event Trial in Type 2 Diabetes Mellitus Patients.

The relative odds for good versus suboptimal risk factor control (ie, 3–4 risk factors versus ≤2 risk factors) in patients with CAD alone was higher than in those with stroke alone across all 3 trials (ORs [95% CI]: CARMELINA 2.05 [1.67–2.51]; EMPA-REG OUTCOME, 2.50 [2.10–2.99]; and CAROLINA, 1.63 [1.21–2.20]; Figure 2). The respective ORs (95% CIs) were lower (and rendered nonsignificant in CAROLINA) when cardiovascular risk factor control in patients with both CAD and stroke were compared with those with stroke alone: CARMELINA, 1.45 (1.13–1.87), EMPA-REG OUTCOME, 1.62 (1.25–2.08), and CAROLINA, 1.16 (0.74–1.83). Using the more stringent criterion of LDL <70 mg/dL or statin use, the results were consistent, showing that the odds for good versus suboptimal risk factor control in patients with CAD alone were higher than those in stroke alone across the 3 trials (ORs [95% CIs]: CARMELINA 2.10 [1.73–2.55]; EMPA-REG OUTCOME 2.44 [2.07–2.89]; and CAROLINA 1.74 [1.31–2.31]). Also, in patients with both CAD and stroke the respective odds for good control were higher than in patients with stroke alone (ORs [95% CIs]: CARMELINA, 1.56 [1.23–1.99], EMPA-REG OUTCOME, 1.65 [1.29–2.10]; and CAROLINA, 1.06 [0.69–1.62]; data not shown). In subgroup analyses by age, sex, and geographic region, the above differences in cardiovascular risk factor control across cardiovascular disease categories were generally consistent in all 3 trials (interaction *P*<0.05), while across regions in EMPA-REG OUTCOME, there was some difference in the magnitude of the effect.

Results for the control of 4 versus less (ie, 0–3) and 2 to 4 versus less (ie, 0–1) cardiovascular risk factors were generally consistent in that the odds of good versus suboptimal control was generally higher in patients with CAD alone versus stroke alone while the differences (odds ratios) were smaller when patients with both CAD and stroke were compared to those with stroke alone (but nonsignificant in CAROLINA; full data shown in Supplemental Material).

Following adjustment for age, sex, and region, the results for good versus suboptimal risk factor control (ie, 3–4 risk factors versus ≤2 risk factors) in patients with CAD alone versus stroke alone and of those with both CAD and stroke versus stroke alone were consistent across all 3 studies (data not shown).

## DISCUSSION

Among 12 856 patients with diabetes in 3 recent cardiovascular outcome trials of glucose-lowering therapy, achievement of goals for preventive therapies appeared better in patients with history of CAD compared with stroke. The largest relative difference in risk factor control was observed between patients with CAD alone versus stroke alone, that is, where clinical management is being influenced by the presence of disease solely in that vascular bed. Our findings were intermediate for those with both CAD and stroke. This may provide a clue for the underlying reasons for the differences we detected. The presence of coronary disease itself, irrespective of stroke, appears to increase the likelihood of better risk factor control. Interestingly, the major driver for the overall difference in risk factor control across the CAD and stroke groups were blood pressure control, LDL-C control, or statin use and use of antiplatelet/anticoagulants which are predominantly clinician-driven. In contrast, there was similar control of smoking across these groups, which is predominantly a patient decision. Although patient adherence plays a role in control of both blood pressure and LDL-C and smoking cessation can indeed be influenced by practitioners, clinician-related factors are likely the major influence in the first 2. The intermediate results in patients with both CAD and stroke support this point. The magnitude of differences was variable across the 3 trials, with the largest differences in the EMPA-REG OUTCOME trial and smallest in the CAROLINA trial. This is likely related to the fact that the latter study’s population generally had more recently diagnosed T2D and fewer underlying comorbidities. According to some studies, the guidelines-adherent management of modifiable cardiovascular risk factors including dyslipidemia, hypertension, and smoking cessation can decrease the risk of recurrence of stroke by about 80%.^7^ Therefore, irrespective of the reasons of these disparities, our findings underscore the need for improved care in patients with stroke.

Ours is the first study conducted in a T2D population enrolled in modern outcome trials, where cardiovascular risk factor control was assiduously recorded. These data are consistent with research in the general population. A multicenter observational case-control study that included 5458 patients from 1444 primary health centers in Spain compared the rates of risk factor control in patients with ischemic stroke versus patients with CAD. The therapeutic targets analyzed in that study were significantly lower in patients after ischemic stroke for hypertension (23.0% versus 27.2%) and dyslipidemia (13.6% versus 20.3%) compared with patients with CAD.^8^ Another retrospective study compared the risk factor control 1 year after discharge in patients with ischemic stroke versus MI and reported higher odds for optimal blood pressure management in patients who suffered MI in the past year compared to those with stroke. However, the study found no differences in control rates for hyperlipidemia and, if anything, higher odds of better glycemic control in patients with ischemic stroke who had T2D.^9^ A third study, involving a nationwide cross-sectional investigation in France, compared the management of hypertension in stroke versus CAD patients and reported poorer blood pressure control in the former versus the latter (24.5% versus 34.2%; *P*<0.01). The study also noted that antihypertensive monotherapy was more common in patients with stroke than in those with CAD (43.2% versus 31.4%; *P*<0.0001), which could explain the differences in blood pressure achievement.^10^

Stroke and CAD are primarily the result of atherosclerosis and treatments for secondary prevention are substantially similar. So why might there be differences in the quality of preventive care for patients with these 2 conditions? One possible explanation is that patients with CAD are more commonly cared for by cardiologists (in addition to primary care providers), who tend to follow their patients regularly and, in many circumstances, indefinitely. This is particularly the case after acute cardiovascular events (eg, MI or invasive coronary interventions). Moreover, this specialty has been particularly focused on risk factor modification. In contrast, however, most patients with stroke will see a neurologist during the hospitalization and later in the office for a limited number of visits. Most patients with stroke may also be seen by physical medicine and rehabilitation providers for several months, but their focus is mainly on the recuperative process with, traditionally, less attention to risk factor assessment or management. The hypothesis that intensity of subspecialty care may explain differences in risk factor control is supported by research from the Veterans Administration showing that patients with history of MI have more outpatient visits (mean of 7.9±6.1 visits [median 7]) compared with patients with history of stroke (mean of 6.0±4.5 visits [median 5]; *P*<0.0001) within the first year after discharge.^9^ The difference was attributable to an increase in cardiology visits.

Other potential explanations for the differences observed relate to underlying disease status (CAD versus stroke). For example, there tends to be an increased prevalence of uncontrolled hypertension in patients with stroke. Data from REGARDS, a population-based cohort study, suggests that the prevalence of apparent resistant hypertension defined as uncontrolled blood pressure with ≥3 antihypertensive medication classes (or use of ≥4 antihypertensive medication classes regardless of blood pressure level) was 24.9% in patients with stroke compared to 17.0% in those without.^11^ Even though the direction of causation between stroke and treatment resistant hypertension, (ie, if the stroke was due to previous resistant hypertension or the resistant hypertension is a result of vascular changes secondary to atherosclerosis) is difficult to establish, a higher percentage of patients with stroke seemed to have difficult to control hypertension based on this study. Also, from these trials’ databases, we were not able to distinguish 2 causes of stroke (ischemic and cardioembolic) which may not respond similarly to modification of all the cardiovascular risk factors analyzed. Finally, guideline-directed therapies in patients with CAD with concurrent heart failure includes renin-angiotensin system blockers, beta blockers, and mineralocorticoid receptor antagonists—each of which have blood pressure–lowering effects. This may contribute to less severe hypertension in at least some patients with CAD who would then be more likely to achieve target blood pressure than in those with stroke. The percentage of patients with control of each cardiovascular risk factor by cardiovascular disease group is shown in Table 3.

**Table 3.**
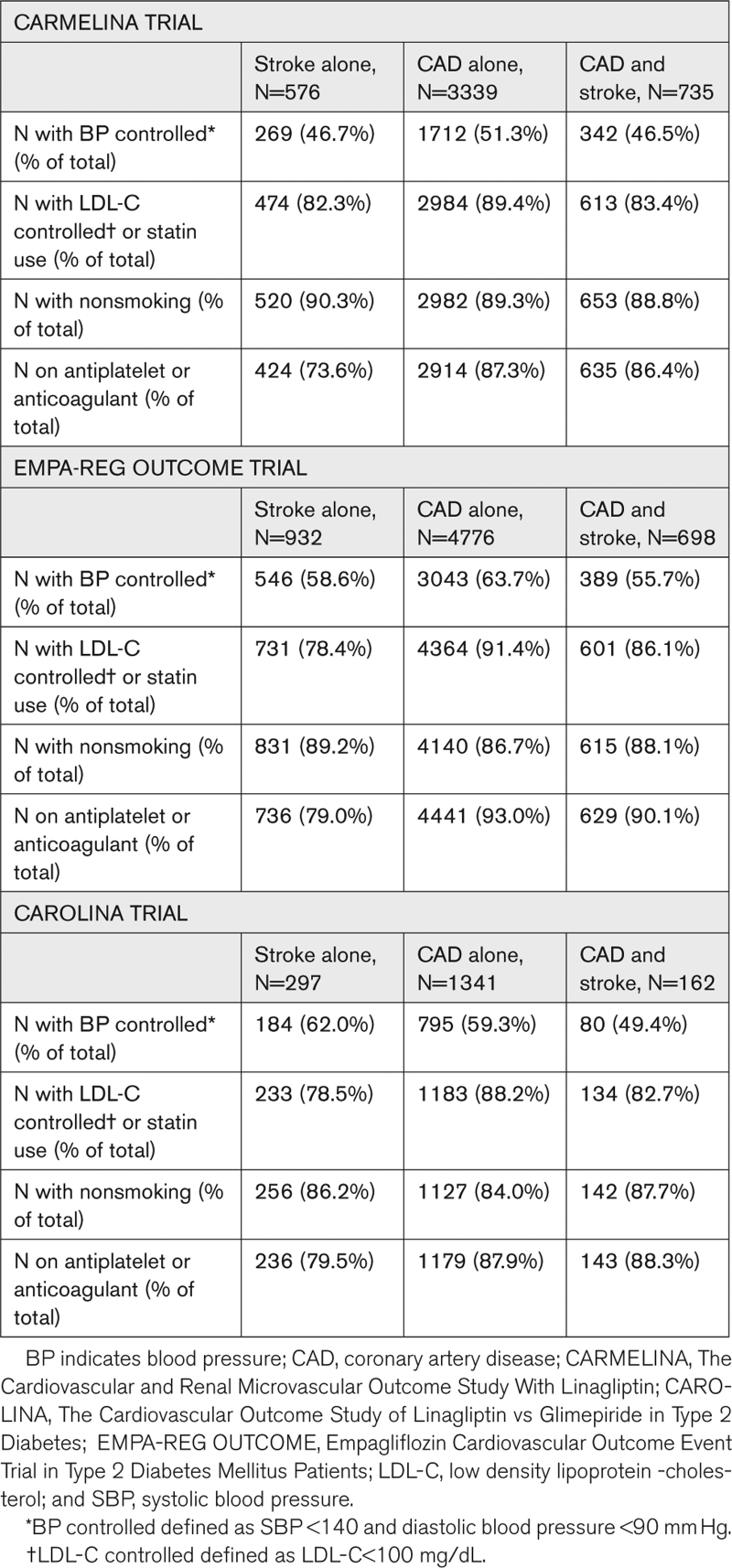
CV Risk Factor Control by CV Disease Groups in the 3 Trials

Patients with more severe strokes may also be vulnerable to clinician implicit bias due to disability and dependency on caregivers for aspects of daily living. This may influence decision-making or perceived treatment goals and contribute to less optimal risk factor management in stroke patients. In the most profoundly affected patients, institutionalization may further limit access to aggressive outpatient care.^12^ In addition, patient-centered communication that is critical to trust and improved treatment adherence could be compromised in patients with residual neurological deficits. Other patient-related factors could also account for the observed differences. Some patients with a history of stroke could have accompanying cognitive impairment either secondary to the stroke itself or prior damage from coexisting cerebrovascular disease, making compliance with medications and follow-up with physician appointments more challenging.

### Limitations

It may be difficult to fully generalize the findings from these 3 cardiovascular outcome trials as their patient populations were dictated by the specific inclusion/exclusion criteria in each. Accordingly, our findings only pertain to the trial populations, and it would be of interest to see how our observations fare in large-scale observational datasets in T2D. We also did not analyze medication data, such as number and class of antihypertensives, use of high/moderate intensity statins to achieve LDL cholesterol level <100 mg/dL, or adherence in the trial participants. These might have provided more insights into the severity of risk factor and provider prescribing patterns. We did not categorize the stroke events as atherosclerotic/lacunar/hemorrhagic strokes. This is a limitation of our study, but we suspect that it would not make a major difference to the results of the study as ischemic stroke is much more common than hemorrhagic strokes in patients with diabetes with only a small fraction having the latter. Finally, our definition of stroke included only those with cerebrovascular events, and not those with cerebrovascular disease, per se. This is in contrast to the definition of CAD in our study, which incorporated both those with prior ischemic events and interventions, but also those with more milder forms of obstructive CAD. Given the systemic nature of atherosclerosis, it is very likely that those individuals assigned to the CAD-only and stroke-only groups actually did have disease in other vascular beds.

### Conclusions

In summary, we found significant discordance in the management of cardiovascular risk factors between patients with stroke versus those with CAD who were enrolled in clinical trials of diabetes therapy. Patients with a history of stroke, compared with CAD, had less optimal risk factor control. The intermediate results in patients with both CAD and stroke suggest that these differences could be related at least in part to clinician factors. Improving clinical outcomes after stroke, particularly as regards to reducing recurrent vascular events, will require a better understanding of the reasons behind these differences. More broadly, addressing them is likely to have a beneficial impact on the health of patients with stroke.

## ARTICLE INFORMATION

Presented in part at the American Diabetes Association Scientific Sessions, New Orleans, LA, June 3–7, 2022.

### Acknowledgments

The authors meet criteria for authorship as recommended by the International Committee of Medical Journal Editors (ICMJE) and were fully responsible for all content and editorial decisions and were involved at all stages of the manuscript development. The authors thank the investigators, coordinators, and patients who participated in the trials. Editorial assistance with preparation of figures was supported financially by Boehringer Ingelheim and provided by Paul Lidbury of Elevate Scientific Solutions.

### Sources of Funding

The EMPA-REG OUTCOME (Empagliflozin Cardiovascular Outcome Event Trial in Type 2 Diabetes Mellitus Patients), CAROLINA (The Cardiovascular Outcome Study of Linagliptin vs Glimepiride in Type 2 Diabetes), and CARMELINA (The Cardiovascular and Renal Microvascular Outcome Study With Linagliptin) trials were sponsored by the Boehringer Ingelheim and Eli Lilly and Company Diabetes Alliance.

### Disclosures

Dr Sheth reports compensation from Astrocyte for consultant services; service as President for Advanced Innovation in Medicine; grants from Hyperfine; employment by Yale School of Medicine; grants from Biogen; compensation from Cerevasc for consultant services; a patent pending for Stroke wearables licensed to Alva Health; compensation from CSL Behring for consultant services; compensation from Sense for data and safety monitoring services; compensation from Certus for consultant services; grants from Novartis; grants from BARD; compensation from ZOLL Medical Corporation for data and safety monitoring services; and compensation from Rhaeos for consultant services. Dr Rosenstock reports grants from Novo Nordisk; compensation from Intarcia for consultant services; travel support from Novo Nordisk; travel support from Applied Therapeutics; grants from Boehringer Ingelheim; travel support from Boehringer Ingelheim; grants from Pfizer; compensation from Oramed for consultant services; grants from Hanmi Pharmaceutical Co, Ltd; compensation from Boehringer Ingelheim for consultant services; compensation from Zealand for consultant services; compensation from Novo Nordisk for consultant services; grants from Intarcia; travel support from Intarcia; compensation from Eli Lilly and Company for consultant services; travel support from Sanofi US Services, Inc; compensation from Regor Pharmaceuticals, Inc for consultant services; compensation from Applied Therapeutics for consultant services; grants from Sanofi US Services, Inc; compensation from Hanmi Pharmaceutical, Co Ltd, for consultant services; grants from Eli Lilly and Company; grants from Novartis; grants from Applied Therapeutics; compensation from Sanofi US Services, Inc for consultant services; grants from Oramed; and travel support from Oramed. Dr Wanner reports compensation from Astellas Pharma for consultant services; compensation from Sanofi US Services, Inc for consultant services; compensation from Fresenius USA, Inc for consultant services; compensation from Idorsia for consultant services; compensation from Vifor Fresenius Medical Care Renal Pharma, Ltd, for consultant services; compensation from Eli Lilly and Company for consultant services; compensation from Amicus Therapeutics Inc. for consultant services; compensation from Takeda California Inc. for consultant services; compensation from Boehringer Ingelheim for consultant services; compensation from Chiesi Farmaceutici for consultant services; compensation from Amgen for consultant services; compensation from GlaxoSmithKline for consultant services; compensation from Merck for consultant services; compensation from AstraZeneca for consultant services; compensation from Novo Nordisk AS for consultant services; compensation from Bayer for consultant services; compensation from Novartis for consultant services; and compensation from Gilead Sciences for consultant services. Dr Zinman has served as a consultant for Boehringer Ingelheim, Novo Nordisk and Eli Lilly. Dr Ofstad and M. Mattheus are employees of Boehringer Ingelheim. Dr Marx reports compensation from AstraZeneca for other services; compensation from Sanofi Aventis for other services; compensation from Boehringer Ingelheim for other services; compensation from Bristol-Myers Squibb for other services; compensation from Bristol-Myers Squibb for other services; compensation from AstraZeneca for consultant services; compensation from Novo Nordisk for other services; compensation from Kowa Research Institute for consultant services; compensation from Novo Nordisk for other services; compensation from Merck Sharp and Dohme for other services; compensation from Genfit for other services; grants from Boehringer Ingelheim; compensation from Boehringer Ingelheim for other services; compensation from Lilly Deutschland for other services; and compensation from Amgen for other services. Dr Inzucchi has served as a consultant and member of clinical trial steering committees for Boehringer Ingelheim, AstraZeneca, Novo Nordisk, Merck, Pfizer, and Bayer. He has delivered lectures sponsored by Boehringer Ingelheim, AstraZeneca, and Merck. The other authors report no conflicts.

### Supplemental Material

Table S1

Figures S1–S2

STROBE checklist

## Supplementary Material



## References

[1] Elnady HM Mohammed GF Elhewag HK Mohamed MK Borai A. Risk factors for early and late recurrent ischemic strokes. Egypt J Neurol Psychiatr Neurosurg. 2020;56:56.

[2] Hardie K Hankey GJ Jamrozik K Broadhurst RJ Anderson C. Ten-year risk of first recurrent stroke and disability after first-ever stroke in the Perth Community Stroke Study. Stroke. 2004;35:731–735. doi: 10.1161/01.STR.0000116183.50167.D9 14764929 10.1161/01.STR.0000116183.50167.D9

[3] Di Angelantonio E Kaptoge S Wormser D Willeit P Butterworth AS Bansal N O’Keefe LM Gao P Wood AM Burgess S; Emerging Risk Factors Collaboration. Association of cardiometabolic multimorbidity with mortality. JAMA. 2015;314:52–60. doi: 10.1001/jama.2015.7008 26151266 10.1001/jama.2015.7008 PMC4664176

[4] Zinman B Wanner C Lachin JM Fitchett D Bluhmki E Hantel S Mattheus M Devins T Johansen OE Woerle HJ; EMPA-REG OUTCOME Investigators. Empagliflozin, cardiovascular outcomes, and mortality in type 2 diabetes. N Engl J Med. 2015;373:2117–2128. doi: 10.1056/NEJMoa1504720 26378978 10.1056/NEJMoa1504720

[5] Rosenstock J Kahn SE Johansen OE Zinman B Espeland MA Woerle HJ Pfarr E Keller A Mattheus M Baanstra D; CAROLINA Investigators. Effect of linagliptin vs glimepiride on major adverse cardiovascular outcomes in patients with type 2 diabetes: the CAROLINA randomized clinical trial: the CAROLINA randomized clinical trial. JAMA. 2019;322:1155–1166. doi: 10.1001/jama.2019.13772 31536101 10.1001/jama.2019.13772 PMC6763993

[6] Hackam DG Spence JD. Combining multiple approaches for the secondary prevention of vascular events after stroke: a quantitative modeling study. Stroke. 2007;38:1881–1885. doi: 10.1161/STROKEAHA.106.475525 17431209 10.1161/STROKEAHA.106.475525

[7] Alvarez-Sabin J Quintana M Hernandez-Presa MA Alvarez C Chaves J Ribo M. Therapeutic interventions and success in risk factor control for secondary prevention of stroke. J Stroke Cerebrovasc Dis. 2009;18:460–465. doi: 10.1016/j.jstrokecerebrovasdis.2009.01.014 19900649 10.1016/j.jstrokecerebrovasdis.2009.01.014

[8] Bravata DM Daggy J Brosch J Sico JJ Baye F Myers LJ Roumie CL Cheng E Coffing J Arling G. Comparison of risk factor control in the year after discharge for ischemic stroke versus acute myocardial infarction. Stroke. 2018;49:296–303. doi: 10.1161/STROKEAHA.117.017142 29284738 10.1161/STROKEAHA.117.017142

[9] Amar J Cambou JP Touzé E Bongard V Jullien G Vahanian A Coppé G Mas J. Comparison of hypertension management after stroke and myocardial infarction: results from ECLAT1--a French nationwide study: results from ECLAT1-a French nationwide study. Stroke. 2004;35:1579–1583. doi: 10.1161/01.STR.0000131547.71502.81 15155960 10.1161/01.STR.0000131547.71502.81

[10] Howard VJ Tanner RM Anderson A Irvin MR Calhoun DA Lackland DT Oparil S Muntner P. Apparent treatment-resistant hypertension among individuals with history of stroke or transient ischemic attack. Am J Med. 2015;128:707–14.e2. doi: 10.1016/j.amjmed.2015.02.008 25770032 10.1016/j.amjmed.2015.02.008 PMC4475646

[11] Kleina T Horn A Suhr R Schaeffer D. Current status of medical care for nursing home residents in Germany - results of an empirical study. Gesundheitswesen. 2017;79:382–387. doi: 10.1055/s-0035-1549971 26110241 10.1055/s-0035-1549971

[12] Arif H Aijaz B Islam M Aftab U Kumar S Shafqat S. Drug compliance after stroke and myocardial infarction: a comparative study. Neurol India. 2007;55:130–135. doi: 10.4103/0028-3886.32783 17558116 10.4103/0028-3886.32783

