# Supplementary material for: Baseline Cardiovascular Risk Factor Control in Patients With Type 2 Diabetes and Coronary Disease Versus Stroke: Secondary Analysis of Cardiovascular Outcome Trials
Source: Stroke. 2023 Jul 14;54(8):2013–21. doi: 10.1161/STROKEAHA.122.042053 (PMC10358436; doi:10.1161/STROKEAHA.122.042053)
Supplement: Supplementary file 1 [file str-54-2013-s001.pdf]

STROBE Statement—Checklist of items that should be included in reports of ***cross-sectional studies***

	Item No	Recommendation	Page
**Title and abstract**	1	(*a*) Indicate the study's design with a commonly used term in the title or the abstract	3 (Abstract)
		(*b*) Provide in the abstract an informative and balanced summary of what was done and what was found	3 (Abstract)
**Introduction**			
Background/rationale	2	Explain the scientific background and rationale for the investigation being reported	5 (Introduction)
Objectives	3	State specific objectives, including any prespecified hypotheses	5 (Introduction)
**Methods**			
Study design	4	Present key elements of study design early in the paper	6 (Methods)
Setting	5	Describe the setting, locations, and relevant dates, including periods of recruitment, exposure, follow-up, and data collection	6 (Methods)
Participants	6	(*a*) Give the eligibility criteria, and the sources and methods of selection of participants	6-7 (Methods)
Variables	7	Clearly define all outcomes, exposures, predictors, potential confounders, and effect modifiers. Give diagnostic criteria, if applicable	7-8, 11-14 (Confounding is addressed in Discussion.)
Data sources/ measurement	8*	For each variable of interest, give sources of data and details of methods of assessment (measurement). Describe comparability of assessment methods if there is more than one group	7-8 (Definition of CV disease groups and CV risk factor control)
Bias	9	Describe any efforts to address potential sources of bias	11-15 (Included in Discussion and Limitations)
Study size	10	Explain how the study size was arrived at	6-9 (Description of design of the studies provided along with references to primary study publications that may be consulted for more details on the event-driven study designs. Statistical Section includes description on population analysed.)
Quantitative variables	11	Explain how quantitative variables were handled in the analyses. If applicable, describe which groupings were chosen and why	7-8 (Definition of CV risk factors)
Statistical methods	12	(*a*) Describe all statistical methods, including those used to control for confounding	8-9 (We do not control for confounding, but this is clarified in our

			discussion and limitations.)
		(*b*) Describe any methods used to examine subgroups and interactions	8-9(Statistical analyses)
		(*c*) Explain how missing data were addressed	8 (Statistical analyses)
		(*d*) If applicable, describe analytical methods taking account of sampling strategy	7-8 (Definition of CV disease groups and CV risk factor control), beyond those no sampling was performed
		(*e*) Describe any sensitivity analyses	8 (Statistical analyses)
**Results**			
Participants	13*	(a) Report numbers of individuals at each stage of study—eg numbers potentially eligible, examined for eligibility, confirmed eligible, included in the study, completing follow-up, and analysed	7-8 (Definition of CV disease groups and CV risk factor control), and Tables, Figures and Suppl. Material provide the number of patients analysed
		(b) Give reasons for non-participation at each stage	7-8 (Definition of CV disease groups and CV risk factor control, Statistical Methods)
		(c) Consider use of a flow diagram	See above where details are given
Descriptive data	14*	(a) Give characteristics of study participants (eg demographic, clinical, social) and information on exposures and potential confounders	Table 2, Suppl. Table 1
		(b) Indicate number of participants with missing data for each variable of interest	7-8 (Definition of CV disease groups and CV risk factor control) Page 8,9 (Statistical analyses) includes the details on which patients were analysed.
Outcome data	15*	Report numbers of outcome events or summary measures	9-10, Table 2, Table 3, Figure 1, Figure 2 and Suppl. Material.
Main results	16	(*a*) Give unadjusted estimates and, if applicable, confounder-adjusted estimates and their precision (eg, 95% confidence interval). Make clear which confounders were adjusted for and why they were included	Unadjusted estimates provided in Result pages 9-10, Table 3, Figure 1, Figure 2 and Suppl. Figure 1 and 2. Sensitivity analyses including adjustment for age, sex, region are described on page 9

			(statistical analyses) and results provided on page 10,11.
		(*b*) Report category boundaries when continuous variables were categorized	7-8 (Definition of CV risk factors)
		(*c*) If relevant, consider translating estimates of relative risk into absolute risk for a meaningful time period	Not applicable
Other analyses	17	Report other analyses done—eg analyses of subgroups and interactions, and sensitivity analyses	Figure 2, Suppl. Figure 1 and 2
**Discussion**			
Key results	18	Summarise key results with reference to study objectives	8-10
Limitations	19	Discuss limitations of the study, taking into account sources of potential bias or imprecision. Discuss both direction and magnitude of any potential bias	14
Interpretation	20	Give a cautious overall interpretation of results considering objectives, limitations, multiplicity of analyses, results from similar studies, and other relevant evidence	11-15
Generalisability	21	Discuss the generalisability (external validity) of the study results	14
**Other information**			
Funding	22	Give the source of funding and the role of the funders for the present study and, if applicable, for the original study on which the present article is based	16

*Give information separately for exposed and unexposed groups.

Note: An Explanation and Elaboration article discusses each checklist item and gives methodological background and published examples of transparent reporting. The STROBE checklist is best used in conjunction with this article (freely available on the Web sites of PLoS Medicine at http://www.plosmedicine.org/, Annals of Internal Medicine at http://www.annals.org/, and Epidemiology at http://www.epidem.com/). Information on the STROBE Initiative is available at www.strobe-statement.org.
