# Supplementary material for: Baseline Cardiovascular Risk Factor Control in Patients With Type 2 Diabetes and Coronary Disease Versus Stroke: Secondary Analysis of Cardiovascular Outcome Trials
Source: Stroke. 2023 Jul 14;54(8):2013–21. doi: 10.1161/STROKEAHA.122.042053 (PMC10358436; doi:10.1161/STROKEAHA.122.042053)
Supplement: Supplementary file 2 [file str-54-2013-s002.pdf]

SUPPLEMENTARY MATERIAL

Supplementary Table (S1) – Baseline medications by CV disease categories in the three trials.

	CARMELINA TRIAL			EMPA-REG OUTCOME TRIAL			CAROLINA TRIAL		
	**Stroke alone n=576**	**CAD alone n=3339**	**CAD and stroke n=735**	**Stroke alone n=932**	**CAD alone n=4776**	**CAD and stroke n=698**	**Stroke alone n=297**	**CAD alone n=1341**	**CAD and stroke n=162**
**Antihypertensives**	**545 (94.6)**	**3264 (97.8)**	**714 (97.1)**	**850 (91.2)**	**4608 (96.5)**	**688 (98.6)**	**269 (90.6)**	**1284 (95.7)**	**158 (97.5)**
Beta-blockers	251 (43.6)	2462 (73.7)	508 (69.1)	278 (29.8)	3575 (74.9)	502 (71.9)	98 (33.0)	973 (72.6)	99 (61.1)
Diuretics	262 (45.5)	1898 (56.8)	398 (54.1)	380 (40.8)	2042 (42.8)	359 (51.4)	112 (37.7)	527 (39.3)	58 (35.8)
ACE inhibitors/ARBs	465 (80.7)	2731 (81.8)	609 (82.9)	722 (77.5)	3885 (81.3)	591 (84.7)	235 (79.1)	1014 (75.6)	131 (80.9)
ACE inhibitors	268 (46.5)	1552 (46.5)	361 (49.1)	439 (47.1)	2490 (52.1)	361 (51.7)	125 (42.1)	671 (50.0)	84 (51.9)
ARBs	205 (35.6)	1248 (37.4)	260 (35.4)	305 (32.7)	1504 (31.5)	253 (36.2)	114 (38.4)	367 (27.4)	50 (30.9)
Calcium channel blocker/antagonists	264 (45.8)	1310 (39.2)	316 (43.0)	384 (41.2)	1479 (31.0)	276 (39.5)	119 (40.1)	444 (33.1)	59 (36.4)
MRAs	22 (3.8)	383 (11.5)	78 (10.6)	31 (3.3)	336 (7.0)	55 (7.9)	6 (2.0)	68 (5.1)	4 (2.5)
Renin inhibitors	0 (0.0)	4 (0.1)	1 (0.1)	10 (1.1)	23 (0.5)	7 (1.0)	3 (1.0)	7 (0.5)	0 (0.0)
Other	78 (13.5)	425 (12.7)	100 (13.6)	88 (9.4)	358 (7.5)	72 (10.3)	22 (7.4)	97 (7.2)	21 (13.0)
**Lipid lowering drugs**	**421 (73.1)**	**2762 (82.7)**	**561 (76.3)**	**643 (69.0)**	**4112 (86.1)**	**567 (81.2)**	**209 (70.4)**	**1148 (85.6)**	**118 (72.8)**
Niacin	0 (0.0)	19 (0.6)	0 (0.0)	4 (0.4)	110 (2.3)	9 (1.3)	1 (0.3)	27 (2.0)	1 (0.6)
Fibrates	39 (6.8)	284 (8.5)	42 (5.7)	61 (6.5)	451 (9.4)	66 (9.5)	11 (3.7)	81 (6.0)	9 (5.6)
Statins	402 (69.8)	2686 (80.4)	547 (74.4)	612 (65.7)	3914 (82.0)	544 (77.9)	199 (67.0)	1099 (82.0)	113 (69.8)
Ezetimibe	6 (1.0)	93 (2.8)	8 (1.1)	17 (1.8)	208 (4.4)	33 (4.7)	8 (2.7)	82 (6.1)	9 (5.6)
Other	9 (1.6)	173 (5.2)	19 (2.6)	27 (2.9)	447 (9.4)	47 (6.7)	12 (4.0)	96 (7.2)	7 (4.3)
**Anti-platelet or anti-coagulant**	**424 (73.6%)**	**2914 (87.3%)**	**635 (86.4%)**	**736 (79.0)**	**4441 (93.0)**	**629 (90.1)**	**236 (79.5%)**	**1179 (87.9%)**	**143 (88.3%)**
Aspirin	322 (55.9)	2520 (75.5)	515 (70.1)	639 (68.6)	4191 (87.8)	563 (80.7)	173 (58.2)	1009 (75.2)	115 (71.0)
Clopidogrel	101 (17.5)	813 (24.3)	170 (23.1)	86 (9.2)	550 (11.5)	90 (12.9)	58 (19.5)	298 (22.2)	31 (19.1)
Dipyridamole	5 (0.9)	3 (0.1)	0 (0.0)	23 (2.5)	11 (0.2)	12 (1.7)	14 (4.7)	5 (0.4)	12 (7.4)
Direct factor XA inhibitors	4 (0.7)	46 (1.4)	20 (2.7)	1 (0.1)	2 (<0.1)	2 (0.3)	1 (0.3)	0 (0.0)	0 (0.0)
Direct thrombin inhibitors	11 (1.9)	25 (0.7)	9 (1.2)	3 (0.3)	14 (0.3)	2 (0.3)	1 (0.3)	4 (0.3)	0 (0.0)
Heparin group	2 (0.3)	28 (0.8)	9 (1.2)	5 (0.5)	11 (0.2)	1 (0.1)	4 (1.3)	3 (0.2)	1 (0.6)
Vitamin K antagonists	34 (5.9)	264 (7.9)	77 (10.5)	40 (4.3)	269 (5.6)	79 (11.3)	24 (8.1)	94 (7.0)	19 (11.7)
Warfarin	18 (3.1)	165 (4.9)	49 (6.7)	13 (1.4)	106 (2.2)	34 (4.9)	13 (4.4)	47 (3.5)	13 (8.0)

All data are n (%)

ACE, angiotensin converting enzyme; ARB, angiotensin receptor blocker; CAD, coronary artery disease; CV, cardiovascular; MRA, mineralocorticoid receptor antagonist.

Supplementary Figure (S1) Forest plot comparing various degrees of CV risk factor control in patients with CAD alone vs. stroke alone.

	Stroke alone		CAD alone		Odds Ratio (95% Confidence interval) of 'CAD alone' vs. 'Stroke alone'	p-value
	n with good control /N at risk	%	n with good control /N at risk	%		
**CARMELINA**						
2–4 vs. 0–1 risk factors controlled	540/576	93.8	3241/3339	97.1	2.21 (1.49, 3.27)	<0.0001
3–4 vs. 0–2 risk factors controlled	413/576	71.7	2800/3339	83.9	2.05 (1.67, 2.51)	<0.0001
4 vs. 0–3 risk factors controlled	159/576	27.6	1217/3339	36.4	1.50 (1.24, 1.83)	<0.0001
**EMPA-REG OUTCOME**						
2–4 vs. 0–1 risk factors controlled	873/932	93.7	4717/4776	98.8	5.41 (3.74, 7.81)	<0.0001
3–4 vs. 0–2 risk factors controlled	714/932	76.6	4257/4776	89.1	2.50 (2.10, 2.99)	<0.0001
4 vs. 0–3 risk factors controlled	329/932	35.3	2239/4776	46.9	1.62 (1.40, 1.87)	<0.0001
**CAROLINA**						
2–4 vs. 0–1 risk factors controlled	282/297	94.9	1298/1341	96.8	1.61 (0.88, 2.93)	0.1230
3–4 vs. 0–2 risk factors controlled	223/297	75.1	1114/1341	83.1	1.63 (1.21, 2.20)	0.0014
4 vs. 0–3 risk factors controlled	107/297	36.0	536/1341	40.0	1.18 (0.91, 1.54)	0.2083

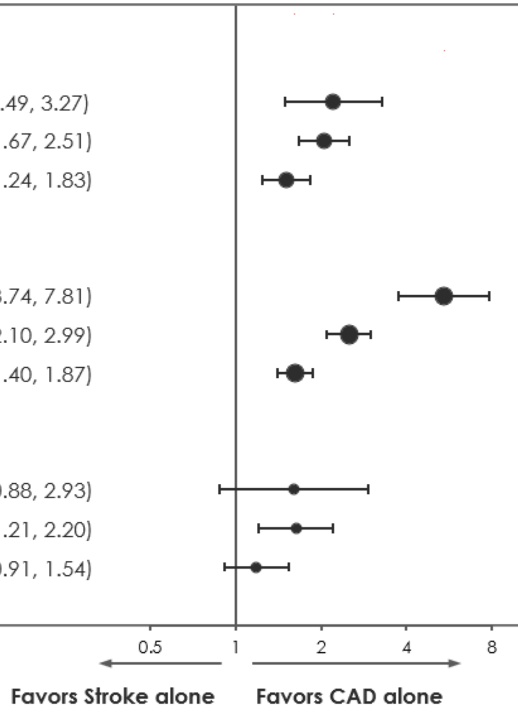

Good control refers to 2–4, 3–4 or 4 factors controlled as indicated per line.
CAD, coronary artery disease; CV, cardiovascular.

Supplementary Figure (S2) Forest plot comparing various degrees of CV risk factor control in patients with CAD and stroke vs. stroke alone.

	Stroke alone		CAD and stroke		Odds Ratio (95% Confidence interval) of 'CAD and stroke' vs. 'Stroke alone'	p-value
	n with good control /N at risk	%	n with good control /N at risk	%		
**CARMELINA**						
2–4 vs. 0–1 risk factors controlled	540/576	93.8	707/735	96.2	1.68 (1.01, 2.79)	0.0438
3–4 vs. 0–2 risk factors controlled	413/576	71.7	578/735	78.6	1.45 (1.13, 1.87)	0.0038
4 vs. 0–3 risk factors controlled	159/576	27.6	225/735	30.6	1.16 (0.91, 1.47)	0.2351
**EMPA-REG OUTCOME**						
2–4 vs. 0–1 risk factors controlled	873/932	93.7	680/698	97.4	2.55 (1.49, 4.37)	0.0006
3–4 vs. 0–2 risk factors controlled	714/932	76.6	587/698	84.1	1.62 (1.25, 2.08)	0.0002
4 vs. 0–3 risk factors controlled	329/932	35.3	270/698	38.7	1.16 (0.94, 1.42)	0.1613
**CAROLINA**						
2–4 vs. 0–1 risk factors controlled	282/297	94.9	152/162	93.8	0.81 (0.36, 1.84)	0.6132
3–4 vs. 0–2 risk factors controlled	223/297	75.1	126/162	77.8	1.16 (0.74, 1.83)	0.5184
4 vs. 0–3 risk factors controlled	107/297	36.0	60/162	37.0	1.05 (0.70, 1.55)	0.8298

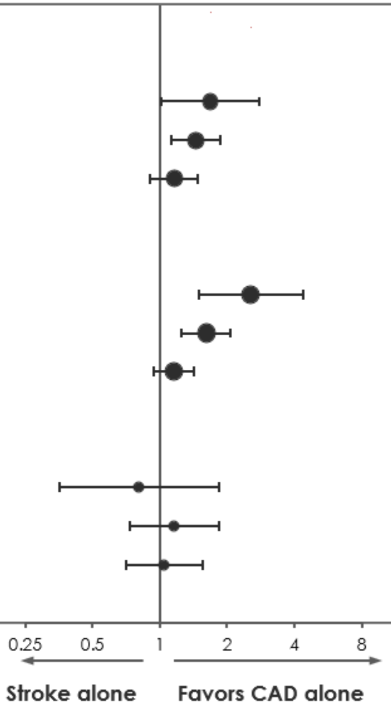

Good control refers to 2–4, 3–4 or 4 factors controlled as indicated per line.
CAD, coronary artery disease; CV, cardiovascular.
